# The Impact of COVID-19 Lockdown on Health Behaviors among Students of a French University

**DOI:** 10.3390/ijerph18084346

**Published:** 2021-04-20

**Authors:** Marie Pierre Tavolacci, Edwin Wouters, Sarah Van de Velde, Veerle Buffel, Pierre Déchelotte, Guido Van Hal, Joel Ladner

**Affiliations:** 1 Clinical Investigation Center, CHU Rouen, U 1073, Normandie University, F 76000 Rouen, France; 2 Faculty of Social Sciences, Department of Sociology Centre for Population, Family & Health, University of Antwerpen, 2180 Antwerpen, Belgium; edwin.wouters@uantwerpen.be (E.W.); Sarah.VandeVelde@uantwerpen.be (S.V.d.V.); Veerle.Buffel@uantwerpen.be (V.B.); 3 Department of Nutrition CHU Rouen, U 1073, Normandie University, F 76000 Rouen, France; pierre.dechelotte@chu-rouen.fr; 4 Department of Epidemiology and Social Medicine University of Antwerpen, 2180 Antwerpen, Belgium; guido.vanhal@uantwerpen.be (G.V.H.); joel.ladner@chu-rouen.fr (J.L.); 5 Department of Epidemiology and Health Promotion, CHU Rouen, U 1073, Normandie University, F 76000 Rouen, France

**Keywords:** COVID-19, lockdown, student, binge drinking, tobacco smoking, depression

## Abstract

This study aimed to determine the changes in health behaviors among students of a French university during the COVID-19 lockdown. An online retrospective survey was distributed to Rouen-Normandy University students at the end of the COVID-19 lockdown (13th March–11th May 2020). Voluntary university students were included. Data collected were on socio-demographics, academic environment, COVID19 concerns, tobacco smoking, binge drinking, cannabis use, and physical activity in the periods before and during COVID-19 lockdown. The survey was completed by 3671 university students (mean age 20.9 ± 2.47 years, 72.9% female). Significantly favorable changes between the periods before and during COVID-19 were reported for tobacco smoking (18.5% vs. 14.8%), binge drinking (35.9% vs. 9.3%) and cannabis use (5.6% vs. 3.2%) and unfavorable changes for moderate (79.4% vs. 67.9%) and vigorous physical activity (62.5% vs. 59.1%). After logistic regression, factors associated with unfavorable changes in tobacco smoking and with favorable changes in vigorous physical activity were the worry of not validating the academic year and stress related to changes in the mode of teaching, respectively. For each health behavior, unfavorable changes were associated with higher depression levels, and male gender. Then as a decrease, mainly in binge drinking was observed during the COVID-19 lockdown, care must be taken to prevent university students from resuming binge drinking after the end of the lockdown. Health-promotion strategies directed at adopting or maintaining positive mental health and promoting physical activity should be developed for university students to better manage future lockdown periods.

## 1. Introduction

On 30 January 2020, the World Health Organization (WHO) declared the coronavirus disease 19 (COVID-19) outbreak a public health emergency of international concern. On 11 March 2020, the WHO Director-General characterized COVID-19 as a pandemic [1]. In March 2020, the outbreak of COVID-19 led the national authorities of most countries in the world to implement extraordinary measures that dramatically restricted the mobility and social interactions of the population, with the aim to limit the transmission of the virus. In France, a lockdown was announced by the President on 16 March, and it entered into force on 17 March, 12:00 CET [2]. Only activities deemed “essential” were maintained, that is, some medical activities, but also food supply, including access to alcohol, as well as tobacco. The lockdown also involved the closure, among others, of universities, and recreational spaces (including sports clubs). The population was required to stay home and only go outside to locations in close proximity for essential needs (including grocery shopping, medical care, legal obligations or limited recreational physical activity within a 1 km radius from home), under police control. Only workers from essential sectors (including healthcare) were allowed to continue their usual activity, with personal protective equipment and physical distancing guidelines. Universities were immediately closed, leading to urgent changes in teaching and examinations, including distance learning without physical class attendance. Despite the fact that the lockdown ended on May 11th in France [3], the universities remained closed with courses and examinations continuing online until the end of the academic year. Ensuring pedagogical continuity required the rapid application of distance learning to the traditional classroom. Some students feared the loss of human contact with an instructor, such as asking questions during and after class, which is known to promote learning, understanding and communication [4]. Many students completing graduate courses face anxiety and panic due to the numerous implications for courses, assignments, seminars and thesis defenses [5]. University students face multiple stressors such as academic overload, constant pressure to succeed, competition with peers and concerns about the future [6]. There have been several reports on the psychological and mental health impact of the COVID-19 outbreak [7,8] and of the lockdown [9,10,11] on university students. Elmer et al. reported that COVID-19 specific concerns, isolation from social networks, lack of interaction and emotional support, and physical isolation were associated with negative mental health among students [12]. Lippi et al. [13] discussed the potential side effects of this stay-at-home policy especially when the lockdown was protracted for months, thereby disrupting social habits and jeopardizing personal health. The most important undesirable effects of a prolonged stay at home are physical inactivity, weight gain, behavioral addiction disorders, insufficient exposure to sunlight, and social isolation [14]. Studies have reported a decrease in physical activity in the general population [15,16] and also in university students [17,18]. Early evidence during the COVID-19 outbreak also suggests negative changes in smoking and alcohol intake associated with higher levels of depression, anxiety, and stress in the general population [19,20] and in teenagers [21]. However, there is limited evidence of the impact of lockdown and teaching disruption on health behaviors among a large sample of university students from different academic curricula.

The objectives of the study, conducted in a sample of French university students during the first wave of restrictions of the COVID−19 pandemic were: To analyze health behaviors during the COVID-19 outbreak and lockdown as tobacco smoking, binge drinking, cannabis use, and physical activity; to assess changes in health behaviors; and to identify factors associated with favorable and unfavorable changes in health behaviors.

## 2. Methods

This study is part of the COVID-19 International Student Well-Being Study (C19 ISWS) [22]. C19 ISWS is the result of a study design, study protocol and questionnaire developed by a team of the University of Antwerp, running at multiple universities during the COVID-19 pandemic [23]. The questionnaire was translated from English to French language through a committee approach (Canada, Switzerland and France). The recruitment strategy thus followed a convenience sampling method. The recruitment goal outlined in the study protocol of the overarching C19 ISWS was to sample at least 10% of the 30,000 student population. University-wide email distribution lists were used to invite students to participate in the study from 13 to 31 May 2020. If students were interested in participating, they were asked to follow a link to the survey website.

### 2.1. Study Design and Participants

This observational study was approved by our Institutional Review Board (E2020-22). All participants provided informed consent to participate in the survey. In order to be eligible to fill out the online questionnaire, study participants had to meet the following criteria: Currently enrolled at a higher education institution, aged 18 years or above, accepting to answer the study questionnaire, willing and able to provide digital informed consent. Participants who had already participated in the study, or who did not understand the language of the questionnaire were not enrolled in the study. Students aged over 30 years were secondarily excluded.

### 2.2. Data Collection

#### Socio Demographic Characteristics

Data were collected on gender, type of academic course (law, literature, healthcare, humanities, and sciences), type of academic degree (bachelor, master, doctorate and if the student was a fresher). Participants were further categorized according to their year of study (year 1, years 2 and 3, years 4 and 5, and year 6 plus). Students reported data on their living accommodation, classified in four groups: (1) Students living with parents before and during COVID-19; (2) students not living with parents before and during COVID-19; (3) students living with parents before but not during COVID-19; and (4) students not living with parents before but living with parents during COVID-19. Students reported whether they had more or less contact with family and friends since the implementation of COVID-19 measures (more, the same or less).

Depression was assessed using the eight items of the CESD-8 (Center for Epidemiologic Studies-Depression) scale [24] and has shown adequate psychometric properties (a Cronbach alpha of 0.82) [24]. The response values are scored on a 4-point Likert scale (range 0 to 3) and CESD-8 on a scale from 0 to 24, with higher scores indicating a higher frequency of depressive complaints.

### 2.3. COVID 19 Lockdown Period and Isolation Measures

#### 2.3.1. Academic Environment

Academic environment was assessed using a Likert scale (1 totally agree to 5 totally disagree) about: Academic stress (increased academic workload, information on university expectations, concern of not being able to validate the academic year, stress with changes in teaching methods) and academic satisfaction (no change in quality of education, sufficiently informed of academic changes, satisfied by the protective measures implemented by the university and being able to talk to the university staff).

#### 2.3.2. COVID-19 Infection

Students were asked whether they had been infected with COVID-19, or were currently infected (yes, confirmed by test; yes confirmed by health care provider; yes I think so, but not confirmed; no), if they knew someone in their personal network that had been or was currently infected with COVID-19. Their concern about COVID-19 was assessed on a scale from 0 to 10: worried about becoming severely ill from a COVID-19 infection; worried about a relative becoming severely ill from a COVID-19 infection; worried about insufficient medical supplies to manage the COVID-19 outbreak.

#### 2.3.3. Health Behaviors

“To assess behaviors before the COVID-19 pandemic, questions were preceded with the phrase “during the month before the COVID-19 measures” and to assess behaviors during the COVID-19 pandemic, questions were preceded with the phrase “during the last week”.

##### Tobacco Smoking, Binge Drinking and Cannabis Use

Students reported, on average, their frequencies of: Tobacco smoking, including cigarettes, cigars, or e-cigarettes, as (almost) never, less than once a week, once a week, (categorized as occasional), more than once a week, and (almost) daily (categorized as regular); binge drinking, defined as six or more glasses of alcohol on a single occasion, as (almost) never, less than once a week (categorized as occasional), once a week, more than once a week and, (almost) daily (categorized as regular); and cannabis use, including marijuana, weed, hash, as (almost) never, less than once a week (categorized as occasional), once a week, more than once a week and, (almost) daily (categorized as regular).

Changes in tobacco smoking, binge drinking and cannabis use were categorized as favorable if, between the periods before and during COVID-19, the frequency changed from occasional or regular to never, or from regular to occasional; as unfavorable if, between the periods before and during COVID-19, the frequency changed from never or occasional to regular, or from never to occasional); and as no change if, between the periods before and during COVID-19, the frequency did not change.

##### Physical Activity

Students reported their frequencies of moderate physical activities, including cycling or walking for at least 30 min, and vigorous physical activities, including lifting heavy weights, running, aerobics, or fast cycling for at least 30 min as (almost) never, less than once a week, once a week (categorized as occasional), more than once a week and (almost) daily (categorized as regular).

Changes in moderate and vigorous physical activity were categorized as favorable if, between the periods before and during COVID-19, the frequency changed from never or occasional to regular, or from never to occasional; as unfavorable if, between the periods before and during COVID-19, the frequency changed from occasional or regular to never, or from regular to occasional; and as no change if, between the periods before and during COVID-19, the frequency did not change.

### 2.4. Statistical Analysis

Participants aged 18 to 30 years, with a completed questionnaire on health behaviors were included. The sample size was estimated as at least 10% of Rouen-Normandy University’s student population (30,000) during a two week period. Among the 4669 participants, 148 were aged 30 years and more. After excluding incomplete questionnaires 3671 questionnaires were included in the analyses. Health behaviors were compared between the periods before and during the COVID-19 outbreak using Student paired t-tests. Multivariable logistic regression model was performed to identify factors associated with favorable and unfavorable changes for each health behavior. Variables with a *p* < 0.20 in univariate analysis were entered in the model. For each health behavior, adjusted odds ratios (aOR) are provided with their 95% confidence interval (95%CI). Interaction terms with gender were tested regarding behavior variables that were included in logistic regression. The three main academic stressors (changes in teaching methods, failing the academic year and an increased workload) were analyzed in univariate and multivariate analysis with two categories: Agree (totally agree and agree) and not agree (no opinion, disagree and totally disagree).

## 3. Results

The intention to sample at least 10% of students was achieved with 3671 university students included (participation rate of 12%). The mean age was 20.9 years (SD = 2.47) and 72.9% were women. Regarding curricula, 36.4% of students were in healthcare, 18.0% in humanities, 13.9% in law, 12.2% in literature, and 19.6% in science. Regarding their personal environment, 69.2% of students were in lockdown with their parents; 26.3% and 50.1% respectively reported less contact with family members and with friends. The mean score of depression based on CESD-8 score was 8.6/24 (ET = 5.1) (Table 1)

### 3.1. Academic Environment

**Figure 1:**
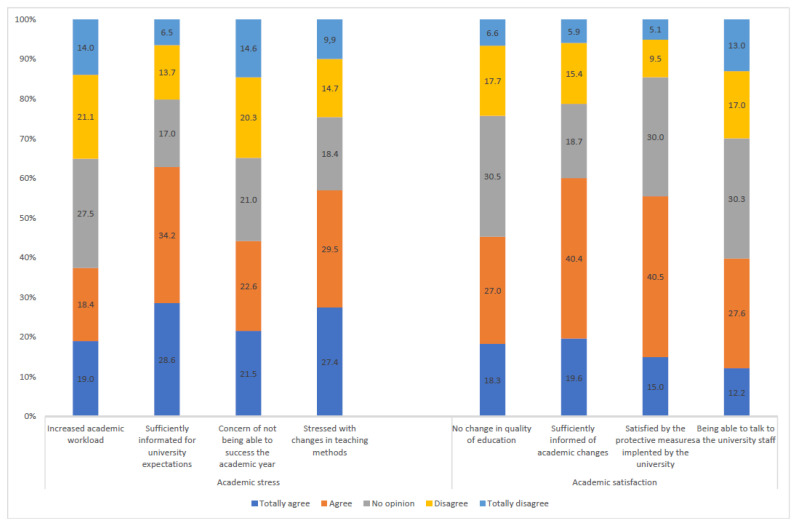
Academic stress and academic satisfaction of the university students (*n* = 3671).

Students’ main concerns were stress due to changes in teaching methods (56.9%), not being able to validate the academic year (44.1%) and an increased workload (37.4%). Students were satisfied with the measures taken by their universities (55.5%) and with communication (60.0%). The responses regarding academic stress and satisfaction are displayed in Figure 1.

### 3.2. COVID-19 Infection

COVID-19 infection was reported by 10.1% of students: 7.8% not confirmed, 2.1% with a medical diagnosis and 0.2% with a laboratory test. The concern of becoming seriously ill was scored 3.6/10 (SD = 3.3); 47.5% of students reported that they knew an infected relative and the concern of a relative being critically ill was scored 7.0/10 (SD = 3.0). The concern of insufficient medical supplies to manage the COVID-19 crisis was scored 6.3/10 (SD = 2.8) (Table 1)

### 3.3. Tobacco Smoking, Binge Drinking, and Cannabis Use

**Figure 2:**
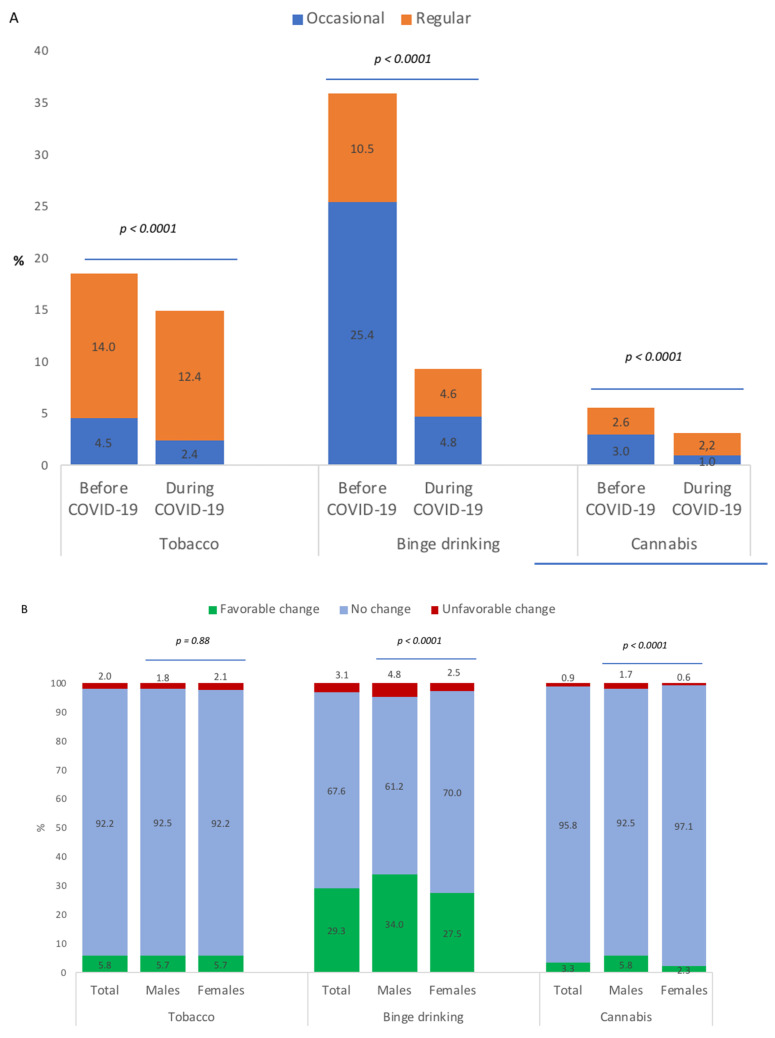
Tobacco smoking, binge drinking and cannabis use of university students before and during COVID-19 (**A**) and changes in behavior between the periods before and during COVID-19 (**B**).

Figure 2 A shows occasional and regular tobacco smoking, binge drinking and cannabis use between the periods before and during COVID-19. Results in Figure 2 (panel A) show a significant decrease in the prevalence of tobacco smoking (18.5% to 14.8%; *p* < 0.001), binge drinking (35.9 to 9.3%; *p* < 0.001), and cannabis use (5.6% to 3.2%; *p* < 0.001) between the periods before and during COVID-19. These decreases represented favorable changes in 5.8% of students for tobacco smoking, in 29.3% for binge drinking, and in 3.3% for cannabis use; and unfavorable changes in 2.0% for tobacco smoking, in 3.1% for binge drinking and in 0.9% for cannabis use (Figure 2 panel B). Favorable and unfavorable changes were more represented in male students than female students for binge drinking and cannabis use.

### 3.4. Factors Associated with Favorable and Unfavorable Changes in Tobacco Smoking, Binge Drinking, and Cannabis Use

Univariate analysis of factors associated with tobacco smoking, binge drinking and cannabis use are displayed in supplementary files (Table S1). After logistic regression, unfavorable changes in tobacco smoking were significantly associated with CESD-8 score and the worry of not being able to validate the academic year. No factor was significantly associated with favorable changes in tobacco smoking. Factors associated with unfavorable changes in binge drinking were male gender, never living with parents and CESD-8 score (Table 2). Factors associated with favorable changes in binge drinking were male gender, healthcare curriculum, year 2 or more of academic curriculum, return to living with parents or staying alone during the COVID-19 outbreak and knowing someone infected by COVID-19 and the fear of becoming seriously ill from a COVID-19 infection (Table 2). Factors associated with unfavorable changes in cannabis use were male gender, return to living with parents or staying alone during the COVID-19 outbreak, and CESD-8 score. Factors associated with favorable changes in cannabis use were male gender and CESD-8 score (Table 2).

### 3.5. Moderate and Vigorous Physical Activity

**Figure 3:**
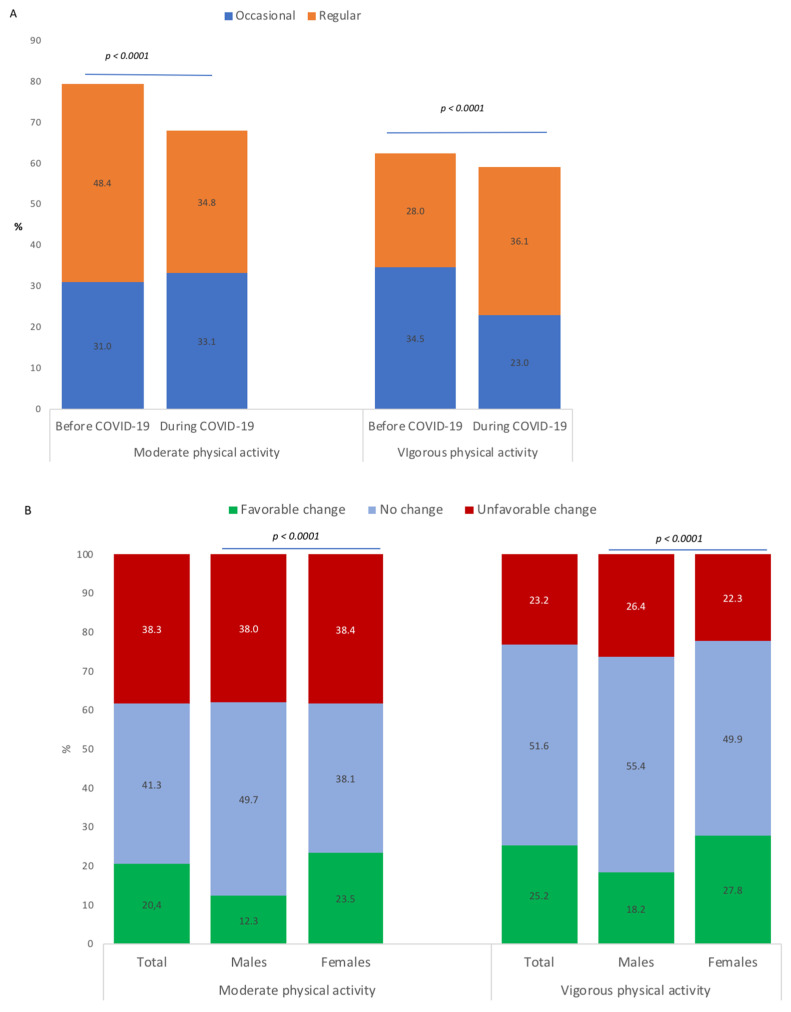
Moderate and vigorous physical activity of university students before and during COVID-19 (**A**) and changes in behavior between the periods before and during COVID-19 (**B**).

Results in Figure 3 (panel A) show a significant decrease in the prevalences of moderate physical activity (79.4 to 67.9%; *p* < 0.001) and vigorous physical activity (62.5% to 59.1%; *p* < 0.001) between the periods before and during COVID-19. These decreases represented favorable changes in 20.4% of students for moderate physical activity and in 25.2% of students for vigorous physical activity; and unfavorable changes in 38.3% of students for moderate physical activity and in 23.2% of students for vigorous physical activity (Figure 3, panel B). Favorable changes were more represented in female students than male students (*p* < 0.001).

### 3.6. Factors Associated with Favorable and Unfavorable Changes in Moderate and Vigorous Physical Activity

For moderate physical activity, unfavorable changes were negatively associated with male gender and positively associated with being personally infected and CESD-8 score. Favorable changes were negatively associated with male gender and positively associated with the fear of becoming seriously ill from a COVID-19 infection (Table 3).

For vigorous physical activity, factors associated with unfavorable changes were not living with parents during the COVID-19 lockdown and CESD-8 score. Favorable changes were negatively associated with male gender and positively associated with stress due to changes in teaching methods (Table 3).

## 4. Discussion

The large sample of different academic curricula allowed us to highlight the effect of the COVID-19 outbreak and isolation measures on university students. We identified concerns about changes in teaching methods, not validating the academic year and increased workload. Specifically, the concern about not validating the academic year was associated with unfavorable changes in tobacco smoking and the concern about changes in teaching methods was associated with favorable changes in vigorous physical activity. To guarantee the effectiveness of online learning, the design principles of digital learning materials, learning goals and students’ preferences and characteristics should be rigorously evaluated [25]. Anxiety due to online learning needs to be relieved to ensure that students can actively and effectively engage in this online learning [26]. In the context of the COVID-19 outbreak, interactive pedagogic tools could be useful for education continuity and for maintaining human contact necessary in pedagogy [4]. Future strategies could combine on-site teaching with online courses and consider the role of social contact in students’ mental health and offer starting points to identify and support students at a higher risk of social isolation and negative psychological effects during the COVID-19 pandemic [12]. The current issue is to safely reopen universities with a combination of strategies that include containment (access control with contact tracing and quarantine) and mitigation (hygiene, sanitation, ventilation, and social distancing) practices [27]. A systematic review showed that medical students undergoing appropriate training could play an essential role in pandemic management and suggests a course and assessment structure for medical student COVID-19 training [28].

In this study, one third of students did not live with their parents and one half had less contact with friends. Bu et al. reported that being a student emerged as a higher risk factor for loneliness during lockdown than usual [29]. Students were worried that their relatives were infected and that medical supplies were lacking as also reported specifically in healthcare students [30]. Studies have suggested that preparation for healthcare students should be provided prior to a pandemic to build resilience, thereby reducing the impact of stress after exposure [31,32].

Unhealthy behaviors (tobacco smoking, binge drinking and cannabis use) significantly decreased during the COVID-19 period especially binge drinking as reported by Busse et al. among German students (ISWS 19 study) [23]. In the general population, studies reported that COVID-19 measures led to increased tobacco smoking [19,33] and that being a student was a protective factor for increased tobacco smoking [19]. The major proportion of favorable changes was for binge drinking, almost one third of students decreased their frequency of binge drinking. This decrease was also reported among Canadian adolescents during the COVID-19 outbreak [21]. Our results, showing that students who returned home to live with their parents during lockdown reduced their practices of binge drinking compared to students living in rented accommodation, support the non-epidemic study [34]. Binge drinking among students revolves around socializing (e.g., partying, and belonging to a social network of heavy drinkers) which may explain why this effect is not found in our study for tobacco and cannabis [34].This decrease of binge drinking was not reported in the general population in the 18–24 year age group [35]. Fear of being infected with COVID-19 was negatively associated with a decrease in binge drinking. Dumas also reported a fear of the infectivity of COVID-19, predicted using solitary substance use during the pandemic [21] and Nguyen et al. found that students with higher fear scores more likely had unhealthy lifestyles, such as smoking and drinking alcohol [36]. In our study, cannabis use decreased both in number of users and frequency. Daumas et al. reported among adolescents a decrease in cannabis use but with an increased frequency [21]. Among the general population, Rolland et al. showed similar rates between an increased use and a decreased use of cannabis and identified that being a student compared to a worker was a protective factor for increased cannabis consumption [32]. We observed that for binge drinking and cannabis use, men had behavioral changes, either better or worse. This can be explained by their mode of consuming before confinement, alone or in a group [21] and by the fact that the men perceived more drinking norms and influence of their peers than women and were therefore more sensitive to the decline of these influences during the lockdown [37].

Concerning positive health behaviors, as physical activity, there were more students with a decrease in physical activity (one third of students) than an increase (one quarter of students) as also reported in studies among students [17,18], and the general population [16]. The role of modifiable lifestyle factors like physical activity in maintaining health and wellness are fundamental [38]. The benefits of empowering students to actively preserve their own health should be underlined [8]. Epidemiological evidence has demonstrated a dose-response relationship between the physical activity performed before infection and a reduction in the incidence, duration, or severity of acute respiratory tract infections [39,40]. The decrease in physical activity due to mandatory stay-at-home is perhaps one of the most apparent consequences of complete lockdown, not only for active individuals habitually practicing recreational sports, but also for those who go to work by walking or cycling. These observations have potential implications that could aid the development of physical activity and nutritional recommendations to maintain health during the ongoing COVID-19 pandemic [15]. Universities have been identified as appropriate settings for the implementation of lifestyle interventions [41].

Depression was associated with an increase in the three unhealthy behaviors and a decrease in physical activity, as reported among adolescent and general populations [19,35]. De Man et al. highlighted in the Belgium C19 ISWS study that the duration of the exposure showed to have an effect on depressive symptoms severity, which was mediated by “academic stress” [42]. A possible mitigation strategy for improving mental health should include taking suitable amounts of daily physical activity and sleeping well [43]. Universities should provide psychological services oriented and adapted to these circumstances to mitigate the emotional impact on university members.

Caution is advised when generalizing these findings, for the following reasons: First, examining changes in health behaviors in the same survey with retrospective measurement of past behavior (before COVID-19 period) may lead to errors in self-reporting; second, it was a convenience sample, voluntary participation could have led to representativeness and self-selection bias as our sample had more women, also reported by an Italian study [18].

## 5. Conclusions

To the authors’ knowledge, this is the first study conducted in a large population of university students in France, focusing on academic teaching disruption, the relationship between health-related behaviors (tobacco smoking, binge drinking cannabis use, and physical activity) during the COVID-19 outbreak and lockdown. These observations have potential implications that could aid the development of physical and nutritional recommendations to maintain health during the ongoing COVID-19 pandemic and specifically target university student populations. Furthermore, at the end of lockdown, care must be taken to prevent university students from resuming binge drinking. The perspectives of the C19-ISWS multicountry study are to target more countries and to collect more responses, allowing a between-country comparison and also a separate analysis of each country’s data to flesh out our results. Future public information campaigns regarding COVID-19 preventions are needed to further emphasize the importance of adopting a healthy lifestyle, especially exercise, during containment.

## Figures and Tables

**Table 1:** Characteristics of university students (*n* = 3671).

	Total
**Gender (*n*)%**	
Male	(995) 27.1
Female	(2676) 72.9
**Curriculum (*n*)%**	
Healthcare	(1336) 36.4
Humanities	(661) 18.0
Law	(510) 13.9
Literature	(448) 12.2
Sciences	(716) 19.5
**Academic year of study (*n*)%**	
1	(1031) 28.1
2 and 3	(1747) 47.6
4 and 5	(716) 19.5
6 and more	(177) 4.8
**Living with parents (*n*)%**	
Before and during C19	(1149) 31.3
Not before and not during C19	(1039) 28.3
Before but not during C19	(92) 2.5
Not before but during C19	(1391) 37.9
**Less contact (*n*)%**	
With family	(965) 26.3
With friends	(1839) 50.1
**CESD-8 scale, mean (SD)**	8.6 (5.1)
**COVID-19**	
Personally infected (*n*)%	(378) 10.1
Knowing others infected (*n*)%	(1748) 47.5
Worried about becoming severely ill, mean (SD)	3.6 (3.3)
Worried about a relative becoming severely ill, mean (SD)	7.0 (3.9)
Worried about insufficient medical supplies, mean (SD)	6.3 (2.8)

CESD-8: Center for Epidemiologic Studies-Depression C19: COVID 19.

**Table 2:** Factors associated with favorable and unfavorable changes in tobacco smoking, binge drinking and cannabis use among university students during the COVID-19 lockdown (*n* = 3671) (Logistic regression).

	Tobacco Smoking		Binge Drinking		Cannabis Use	
	Favorable Change AOR (95% CI)	Unfavorable Change AOR (95% CI)	Favorable Change AOR (95% CI)	Unfavorable Change AOR (95% CI)	Favorable Change AOR (95% CI)	Unfavorable Change AOR (95% CI)
Female			Ref	Ref	Ref	Ref
Male			1.52 (1.29–1.79)	2.24 (1.51–3.33)	3.10 (2.11–4.53)	3.35 (1.65–6.78)
**Curriculum**						
Healthcare			1.29 (1.02–1.64)	0.71 (0.39–1.28)	0.80 (0.43–1.46)	0.46 (0.16–1.30)
Humanities			0.95 (0.72–1.25)	0.72 (0.37–1.40)	0.98 (0.50–1.93)	0.75 (0.24–2.31)
Law			Ref	Ref		
Literature			1.06 (0.78–1.42)	1.18 (0.62–2.27)	1.85 (0.98–3.51)	1.17 (0.40–3.42)
Sciences			0.82 (0.63–1.08)	0.72 (0.38–1.36)	0.69 (0.35–1.36)	0.53 (0.17–1.64)
**Academic year of study**						
1	Ref	Ref	Ref	Ref		
2 and 3	1.20 (0.86–1.69)	1.47 (0.81–2.65)	1.64 (1.36–1.97)	1.25 (0.78–1.99)		
4 and 5	1.13 (0.73–1.75)	0.64 (0.27–1.52)	1.81 (1.44–2.29)	1.16 (0.65–2.09)		
6 and more	0.69 (0.28–1.67)	0.60 (0.13–2.62)	1.51 (1.04–2.20)	0.17 (0.02–1.28)		
**Living with parents**						
Before and during C19	Ref	Ref	Ref	Ref	Ref	
Not before and not during C19	0.78 (0.52–1.19)	1.78 (0.98–3.24)	1.33 (1.08–1.63)	2.30 (1.39–3.80)	1.21 (0.74–1.99)	2.63 (1.08–6.42)
Before but not during C19	0.42 (0.10–1.76)	2.57 (0.84–7.88)	1.13 (0.67–1.88)	1.05 (0.24–4.54)	1.04 (0.24–4.45)	9.95 (2.78–35.77)
Not before but during C19	1.36 (0.98–1.89)	0.79 (0.41–1.51)	1.88 (1.57–2.26)	1.36 (0.81–2.27)	1.37 (0.87–2.18)	0.82 (0.27–2.47)
**Less contact**						
With family			0.83 (0.69–1.02)	0.98 (0.57–1.71)		
With friends			1.15 (0.92–1.45)	0.60 (0.35–1.02)		
**CESD-8 scale**	1.01 (0.98–1.04)	1.07 (1.02–1.12)	0.99 (0.98–1.01)	1.04 (1.00–1.08)	1.05 (1.01–1.09)	1.10 (1.03–1.17)
**COVID-19**						
Personally infected	0.82 (0.49–1.36)	1.69 (0.90–3.19)				
Knowing others infected			1.26 (1.09–1.47)	1.94 (1.31–2.87)		
Worried about becoming severely ill			0.96 (0.94–0.98)	0.95 (0.90–1.02)		
Worried about a relative becoming severely ill						
Worried about insufficient medical supplies						
**University**						
Increased academic workload	0.76 (0.53–1.10)	0.92 (0.53–1.62)	1.13 (0.94–1.36)	0.87 (0.55–1.37)		
Worried about not completing the academic year	1.00 (0.67–1.48)	2.74 (1.14–6.58)	1.00 (0.82–1.22)	1.10 (0.67–1.83)		
Stressed with changes in teaching methods	1.14 (0.76–1.71)	1.03 (0.53–2.01)				

C19: COVD19 lockdown CESD-8: Center for Epidemiologic Studies Depression; Interaction gender and variables tested: *p* > 0.05.

**Table 3:** Factors associated with favorable and unfavorable changes in moderate and vigorous physical activity among university students during the COVID-19 lockdown (*n* = 3671).

	Moderate Physical Activity		Vigorous Physical Activity	
	Favorable Change AOR (95% CI)	Unfavorable Change AOR (95% CI)	Favorable Change AOR (95% CI)	Unfavorable Change AOR (95% CI)
Male Gender	0.41 (0.33–51)	0.77 (0.65–0.90)	0.61 (0.50–0.74)	1.10 (0.92–1.32)
**Curriculum**				
Healthcare			1.07 (0.83–1.38)	1.20 (0.92–1.56)
Humanities			0.88 (0.66–1.17)	0.86 (0.63–1.16)
Law			Ref	Ref
Literature			1.01 (0.74–1.37)	0.77 (0.55–1.09)
Sciences			0.84 (0.63–1.12)	1.01 (0.76–1.35)
**Academic year of study**				
1		Ref	Ref	Ref
2 and 3	0.93 (0.76–1.15)	1.11 (0.93–1.33)	1.00 (0.83–1.21)	1.17 (0.96–1.43)
4 and 5	0.83 (0.63–1.09)	1.07 (0.86–1.35)	1.02 (0.79–1.37)	1.13 (0.88–1.47)
6 and more	0.72 (0.44–1.18)	1.06 (0.74–1.54)	0.64 (0.40–1.02)	1.01 (0–76-1.35)
**Living with parents**				
Before and during C19		Ref	Ref	
Not before and not during C19	0.90 (0.70–1.16)	1.27 (0.93–1.33)	0.84 (0.67–1.05)	1.38 (1.11–1.73)
Before but not during C19	1.11 (0.62–1.98)	1.01 (0.86–1.35)	0.89 (0.50–1.59)	1.79 (1.08–2.98)
Not before but during C19	1.11 (0.90–1.37)	1.02 (0.74–1.54)	1.19 (0.99–1.44)	1.13 (0.92–1.39)
**Less contact**				
With family	0.82 (0.64–1.06)	0.90 (0.78–1.17)	0.85 (0.68–1.06)	1.02 (0.81–1.28)
With friends	0.97 (0.73–1.28)	1.02 (0.80–1.28)	0.99 (0.77–1.27)	1.02 (0.82–1.28)
**CESD-8 scale**	0.99 (0.97–1.00)	1.05 (1.01–1.07)	0.98 (0.97–1.00)	1.03 (1.01–1.05)
**COVID-19**				
Personally infected	1.18 (0.87–1.60)	1.28 (1.00–1.64)	1.09 (0.82–1.42)	1.34 (1.02–1.74)
Knowing others infected			1.15 (0.98–1.34)	1.09 (0.92–1.28)
Worried about becoming severely ill	1.04 (1.01–1.07)	1.01 (0.99–1.03)		
Worried about a relative becoming severely ill	1.02 (0.99–1.05)	1.00 (0.97–1.02)	1.01 (0.98–1.04)	1.00 (0.97–1.03)
Worried about insufficient medical supplies	0.99 (0.96–1.03)	0.01 (0.98–1.03)	1.02 (0.99–1.05)	1.01 (0.98–1.05)
**University**				
Increased academic workload	0.92 (0.73–1.16)	1.02 (0.85–1.23)	0.84 (0.69–1.03)	1.08 (0.88–1.33)
Worried about not completing the academic year	0.87 (0.69–1.10)	1.12 (0.91–1.36)	0.83 (0.67–1.03)	1.03 (0.82–1.29)
Stressed with changes in teaching methods	1.01 (0.79–1.29)	1.07 (0.87–1.32)	1.50 (1.20–1.88)	1.26 (1.00–1.59)

CESD-8: Center for Epidemiologic Studies-Depression.

## Data Availability

Data are available on request.

## Supplementary Materials

The following are available online at https://www.mdpi.com/article/10.3390/ijerph18084346/s1, Table S1: Univariate Analysis.
