# Supplementary material for: The Impact of COVID-19 Lockdown on Health Behaviors among Students of a French University
Source: Int J Environ Res Public Health. 2021 Apr 20;18(8):4346. doi: 10.3390/ijerph18084346 (PMC8072635; doi:10.3390/ijerph18084346)
Supplement: Supplementary file 1 [file ijerph-18-04346-s001.zip › ijerph-1167727-supplementary.pdf]

Table S1. Univariate Analysis.

Tobacco smoking	Favorable change	No change	Unfavorable change	p
**Male Gender**	26.9	27.2	24.6	0.64
**Courses**				0.71
Healthcare	37.7	36.4	34.2	
Humanities	17.5	17.9	21.9	
Law	16.0	13.8	12.3	
Literature	13.7	12.0	15.1	
Sciences	15.1	19.9	16.4	
**Academic year of study**				0.14
1	26.4	28.4	21.9	
2 and 3	51.4	47.0	63.0	
4 and 5	18.9	19.7	12.3	
6 and more	3.3	5.0	2.7	
**Living with parents**				0.002
Before and;during C19	30.0	31.4	27.4	
Not before and not during C19	21.4	28.4	41.1	
Before but not during C19	1.0	2.5	5.5	
Not before but during C19	47.6	37.6	26.0	
**Less contact**				
With family	23.0	26.4	29.2	0.69
With friends	48.8	50.2	45.8	0.90
**CESD scale**	8.5 (4.8)	8.6 (5.1)	11.0 (5.7)	0.001
**COVID-19**				
Personally infected	8.6	10.0	16.4	0.15
Knowing others infected	49.5	47.2	53.4	0.53
Worried about becoming severely ill	3.5 (3.1)	3.6 (3.3)	3.6 (3.3)	0.92
Worried about a relative becoming severely ill	6.9 (2.8)	7.1 (3.0)	7.3 (2.8)	0.29
Worried about insufficient medical supplies	6.1 (2.8)	6.4 (2.8)	6.7 (2.5)	0.22
**University**				
Increased of academic workload	31.1	37.6	44.4	0.13
Concern of not completing the academic year	40.7	44.0	59.7	0.02
Stressed with change in teaching methods	55.5	56.8	69.4	0.17

Binge drinking	Favorable change	No change	Unfavorable change	p
**Male Gender**	31.4	24.5	42.1	<0.0001
**Courses**				0.0003
Healthcare	42.1	34.4	27.2	
Humanities	16.9	18.5	16.7	
Law	12.5	14.3	16.7	
Literature	11.8	12.1	18.4	
Sciences	16.7	20.7	21.1	
**Academic year of study**				<0.0001
1	21.2	31.4	24.6	
2 and 3	50.9	45.9	52.6	
4 and 5	22.4	18.0	21.9	
6 and more	5.5	4.7	0.9	
**Living with parents**				<0.0001
Before and;during C19	24.0	34.7	25.0	
Not before and not during C19	28.3	27.7	41.1	
Before but not during C19	2.2	2.6	1.8	
Not before but during C19	45.5	34.9	32.1	
**Less contact**				
With family	26.8	26.2	22.5	0.05
With friends	53.9	48.7	44.1	0.009

**CESD scale**	8.5 (4.9)	8.6 (5.2)	9.7 (5.6)	0.11
**COVID-19**				
Personally infected	10.1	10.2	7.1	0.57
Knowing others infected	50.3	45.7	59.8	0.004
Worried about becoming severely ill	3.3 (3.1)	3.8 (3.4)	3.2 (3.1)	0.002
Worried about a relative becoming severely ill	7.1 (2.8)	7.0 (3.0)	6 .((3.3)	0.35
Worried about insufficient medical supplies	6.2 (2.8)	6.4 (2.8)	6.4 (2.8)	0.34
**University**				
Increased of academic workload	37.5	37.2	39.3	0.11
Concern of not completing the academic year	42.5	44.4	51.8	0.19
Stressed with change in teaching methods	55.8	57.2	60.7	0.34
**Cannabis use**	**Favorable change**	**No change**	**Unfavorable change**	**p**
**Male Gender**	49.2	26.1	50.0	<0.0001
**Courses**				0.02
Healthcare	29.2	36.8	23.5	
Humanities	18.3	18.0	17.6	
Law	13.3	13.8	20.6	
Literature	22.5	11.7	20.6	
Sciences	16.7	19.7	17.6	
**Academic year of study**				0.34
1	24.2	28.3	23.5	
2 and 3	56.7	47.1	52.9	
4 and 5	15.8	19.6	23.5	
6 and more	3.3	4.9	0.0	
**Living with parents**				<0.001
Before and;during C19	28.0	31.5	20.6	
Not before and not during C19	29.7	28.0	50.0	
Before but not during C19	1.7	2.4	11.8	
Not before but during C19	40.7	38.0	17.6	
**Less contact**				
With family	22.9	26.3	29.4	0.30
With friends	58.5	49.8	52.9	0.30
**CESD scale**	9.7 (5.2)	8.5 (5.0)	11.3 (5.4)	<0.001
**COVID-19**				
Personally infected	12.7	9.9	14.7	0.41
Knowing others infected	52.5	47.3	52.9	0.72
Worried about becoming severely ill	3.2 (3.2)	3.6 (3.3)	4.4 (3.9)	0.29
Worried about a relative becoming severely ill	6.9 (3.0)	7.0 (3.0)	7.0 (3.5)	0.90
Worried about insufficient medical supplies	6.0 (2.9)	6.4 (2.8)	6.6 (2.8)	0.43
**University**				
Increased of academic workload	38.1	37.2	44.1	0.92
Concern of not completing the academic year	45.8	43.9	50.0	0.53
Stressed with change in teaching methods	60.2	56.6	73.5	0.33
**Moderate physical activity**	**Favorable change**	**No change**	**Unfavorable change**	**p**
Table 4	Favorable change	No change	Unfavorable change	p
**Male Gender**	16.3	32.6	26.8	<0.0001
**Courses**				0.47
Healthcare	13.4	14.0	14.1	
Humanities	34.1	35.8	38.3	
Law	19.9	18.5	16.5	
Literature	11.6	12.6	12.0	
Sciences	20.9	19.2	19.1	
**Academic year of study**				0.04
1	31.5	28.6	25.9	
2 and 3	48.1	46.5	48.4	
4 and 5	17.1	19.7	20.5	
6 and more	3.3	5.2	5.3	
**Living with parents**				<0.0001

Before and;during C19	31.5	32.8	29.5	
Not before and not during C19	22.6	27.2	32.5	
Before but not during C19	2.8	2.5	2.3	
Not before but during C19	43.1	37.5	35.7	
**Less contact**				
With family	24.1	25.3	28.5	0.03
With friends	47.6	48.1	53.5	0.004
**CESD scale**	8.0 (4.8)	8.1 (5.0)	9.6 (5.1)	<0.0001
**COVID-19**				
Personally infected	10.6	8.6	11.3	0.04
Knowing others infected	45.4	48.0	48.1	0.19
Worried about becoming severely ill	3.9 (3.5)	3.5 (3.3)	3.6 (3.2)	0.04
Worried about a relative becoming severely ill	7.2 (2.9)	6.9 (3.0)	7.1 (2.9)	0.03
Worried about insufficient medical supplies	6.4 (2.8)	6.3 (2.9)	6.4 (2.8)	0.76
**University**				
Increased of academic workload	35.6	36.0	39.8	0.06
Concern of not completing the academic year	41.3	41.7	48.3	0.001
Stressed with change in teaching methods	58.5	53.6	59.7	0.003

Vigorous physical activity	Favorable change	No change	Unfavorable change	p
**Male Gender**	19.6	29.0	30.6	<0.0001
**Courses**				0.02
Healthcare	37.7	34.3	39.7	
Humanities	17.8	18.9	16.4	
Law	14.5	13.7	13.7	
Literature	13.3	12.7	9.7	
Sciences	16.7	20.4	20.6	
**Academic year of study**				0.04
1	30.0	28.7	24.9	
2 and 3	48.0	46.7	48.9	
4 and 5	18.9	19.3	20.4	
6 and more	3.1	5.2	5.7	
**Living with parents**				<0.0001
Before and;during C19	31.6	32.7	27.8	
Not before and not during C19	22.8	28.4	33.9	
Before but not during C19	2.0	2.4	3.3	
Not before but during C19	43.6	36.5	35.1	
**Less contact**				
With family	24.7	25.6	29.4	0.11
With friends	49.5	48.1	55.1	0.004
**CESD scale**	8.2 (4.7)	8.4 (5.1)	9.5 (5.3)	<0.0001
**COVID-19**				
Personally infected	9.9	9.2	12.1	0.07
Knowing others infected	50.4	46.0	47.8	0.0001
Worried about becoming severely ill	3.8 (3.4)	3.5 (3.3)	3.6 (3.2)	0.21
Worried about a relative becoming severely ill	7.2 (3.0)	6.9 (3.0)	7.1 (2.8)	0.13
Worried about insufficient medical supplies	6.5 (2.8)	6.2 (2.9)	6.5 (2.8)	0.06
**University**				
Increased of academic workload	35.0	36.3	42.3	0.006
Concern of not completing the academic year	41.9	43.0	49.2	0.007
Stressed with change in teaching methods	60.4	53.3	61.2	0.003

C19 : COVID-19 ; CESD : Center for Epidemiologic Studies-Depression
